# The association between striae gravidarum and perineal lacerations during labor

**DOI:** 10.1371/journal.pone.0265149

**Published:** 2022-03-15

**Authors:** Tamar Matyashov, Ella Pardo, Reut Rotem, Yael Lichtman, Maayan Elnir Katz, Adi Y. Weintraub, Amir Horev

**Affiliations:** 1 Department of Obstetrics and Gynecology, Soroka University Medical Center, Ben-Gurion University of the Negev, Beer-Sheva, Israel; 2 Ben-Gurion University of the Negev, Beer-Sheva, Israel; 3 Department of Obstetrics and Gynecology, Shaare Zedek Medical Center, Affiliated with the Hebrew University Medical School of Jerusalem, Jerusalem, Israel; 4 Pediatric Dermatology Services, Soroka University Medical Center, Ben-Gurion University of the Negev, Beer-Sheva, Israel; Affiliated Hospital of Jiangsu University, CHINA

## Abstract

**Objective:**

Striae gravidarum (SG) and perineal lacerations are common occurrences during late pregnancy and labor. It has been hypothesized that both conditions may share a common pathophysiological pathway through changes in the connective tissue. We aimed to investigate a possible association between these two conditions and whether the presence of SG may predict perineal lacerations.

**Methods:**

We conducted a prospective cohort study that included women who gave birth at the Soroka University Medical Center (SUMC), Beer-Sheva, Israel. Those who provided informed consent were examined for the presence of SG using the Davey scoring system to determine the severity of abdominal SG. Clinical and obstetrical characteristics and the presence and degree of perineal tears were retrieved from the computerized patients’ records. Univariate analysis was carried using appropriate statistical tests.

**Results:**

A total of 187 women were recruited. Of those, 81 (43.3%) did not have SG, 24 (12.8%) 43 (23%) and 39 (20.9%) had mild, moderate and severe SG, respectively. Women with SG were significantly older and had a higher body mass index (p<0.01 for both). Delivery characteristics, mode of delivery, and gestational age were comparable between the groups; however, women with SG gave birth to significantly larger neonates (p<0.01). Seventy-one (31%) women had suffered from 1^st^ or 2^nd^-degree perineal tears, and none had 3^rd^ or 4^th^-degree perineal tears. No significant differences were found in rates of perineal tears between women with and without SG (p = 0.91), regardless of SG severity (p = 0.38).

**Conclusions:**

In our study, SG was not associated with perineal tears. This information may be used as reassurance when giving antepartum consultation to women with SG, even in severe cases.

## Introduction

Striae distensae or "stretch marks" are referred to as striae gravidarum (SG) when they occur during pregnancy. SG is a common skin problem reported to affect 55% to 90% of gravid women [1, 2]. They are defined as atrophic linear scars in dermal stretching areas, most commonly the abdomen, breasts, buttocks, hips, and thighs. They appear most commonly in the second half of pregnancy after the 24^th^ week of gestation [3]. Little is known regarding the pathophysiology of SG. Nevertheless, genetic factors (such as chronic genetic diseases and family history) and physical factors (such as elevated pregestational body mass index (BMI), excess weight gain during pregnancy, large for gestational age fetuses) have been shown to contribute to this bothersome condition [1, 3, 4]. These skin lesions disrupt the affected women’s quality of life (QoL) as they are considered aesthetically undesirable and therefore pose a significant psychosocial and therapeutic challenge [5]. Few scoring systems are used to evaluate the severity of SG. One of the more common scoring systems is the Davey Scoring System. In Davey’s method, SG distribution over the entire abdomen is evaluated. The assessment method is simple and easy to perform, leaving little room for error, thus making it widely used [6, 7].

One of the most common complications of delivery is perineal trauma. Perineal trauma is associated with short- and long-term maternal complications such as bleeding, hematoma, infection, abscess formation, the need to suture, urine and fecal incontinence, weakening pelvic floor muscles, dyspareunia, and persistent perineal pain. These may impact many aspects of the parturients’ life, including the mother-baby interaction, breastfeeding, postpartum mood, sexual activity, and postpartum physical and emotional recovery, significantly impacting women’s QoL [8, 9]. More than 60% of women suffer from perineal trauma following a spontaneous vaginal delivery, most of them needing perineal repair [9].

An association between SG and the presence and severity of perineal lacerations during labor has been suggested [10]. It has been hypothesized that the connective tissue of women prone to SG undergoes an abnormal reaction to the physiological stretch during pregnancy and labor. Pregnancy, confers unique alterations to the mechanical properties of connective tissues in order to meet their physiological demands [11] Studies that demonstrated a slower than normal outgrowth of fibroblasts in the skin from SG and a reduced level of fibrillin, collagen and elastin support this hypothesis [12, 13].

The association between SG and perineal tears during labor has been scarcely studied. Therefore, we hypothesized that both SG and perineal lacerations share a common pathophysiological pathway through changes in the connective tissue. In this study, we aimed to investigate a possible association between the presence of SG and the occurrence of perineal trauma.

## Materials and methods

A prospective cohort study was conducted at the Soroka University Medical Center (SUMC) between April 2018 and June 2020. SUMC is a tertiary hospital and the sole medical facility which provides obstetrical admission and delivery services for all residents of the Negev, the southern district of Israel. It is estimated that 98% of deliveries in the district take place at SUMC [1], The study aimed to evaluate the possible association between SG and perineal lacerations, and as such primary outcome was defined as the presence of 1^st^- and 2^nd^-degree perineal tears. A secondary outcome was defined as the presence of 3^rd^- and 4^th^-degree perineal tears. Patients were randomly recruited in the obstetrical emergency room (ER). The Davey scoring system was used to evaluate the severity of SG [14] According to this system, the pregnant patient’s abdomen was divided into four quadrants by two perpendicular lines crossing the umbilicus. Each quadrant receives a score according to the severity of its striae: 0 –no SG, 1 –one SG that is limited to the quadrant, 2 –two or more SG that are limited to the quadrant, or SG that involves more than one quadrant. Mild SG was defined as those who received 1–3, moderate SG 4–6, and severe SG 7–8.

Women over 18 years with a singleton pregnancy during their third trimester were included in the study. Women in active labor, women with a planned elective cesarean section, a known connective tissue disease, known Cushing’s syndrome, women with polyhydramnios (defined as amniotic fluid index ≥25 mm or maximal vertical pocket ≥ 8mm) were excluded from the study. After providing a written informed consent, participants were examined for SG by the research staff during routine fetal monitoring prior to their examination by the physician. Later, when patients gave birth, the presence and degree of perineal tears were retrieved from the computerized patient’s records registered in SUMC. Additionally, demographical, clinical and obstetrical data were also retrieved from the institutional computerized patient’s records. These patients were divided into two groups: those with SG (study group) and those without SG (control group). The collected data were coded and stored using a Microsoft Excel program and then analyzed using SPSS 23.0 (SPSS, Chicago, IL). The initial analysis was performed using descriptive statistics (mean, SD, graphs), followed by advanced analytical statistics using the appropriate tests. First, continuous variables with normal distribution were presented as mean±SD and compared using the t-test. Next, continuous variables that are not normally distributed were presented as median with inter-quartile range, and their statistical analysis was performed using the Mann-Whitney test. Next, categorical variables were presented in counts and percentages, and their statistical analysis was performed using Chi-Square or Fisher Exact test when appropriate. All analyses with a two-sided p-value <0.05 were considered significant. The study received the institutional ethical review board approval (IRB SOR- 0452171).

We have conducted a sample size calculation according to a previous study that examined the association between SG and perineal lacerations [15]. Assuming the prevalence of perineal lacerations in the different groups will be similar in our study and given an alpha of 0.05 and a power of 0.8, the required sample size was 182 participants. Calculation was performed using the WinPepi program. Considering a 17% prevalence of cesarean deliveries in our medical center and another 8%-10% loss to follow-up we decided to recruit 230 patients.

## Results

**Fig 1:**
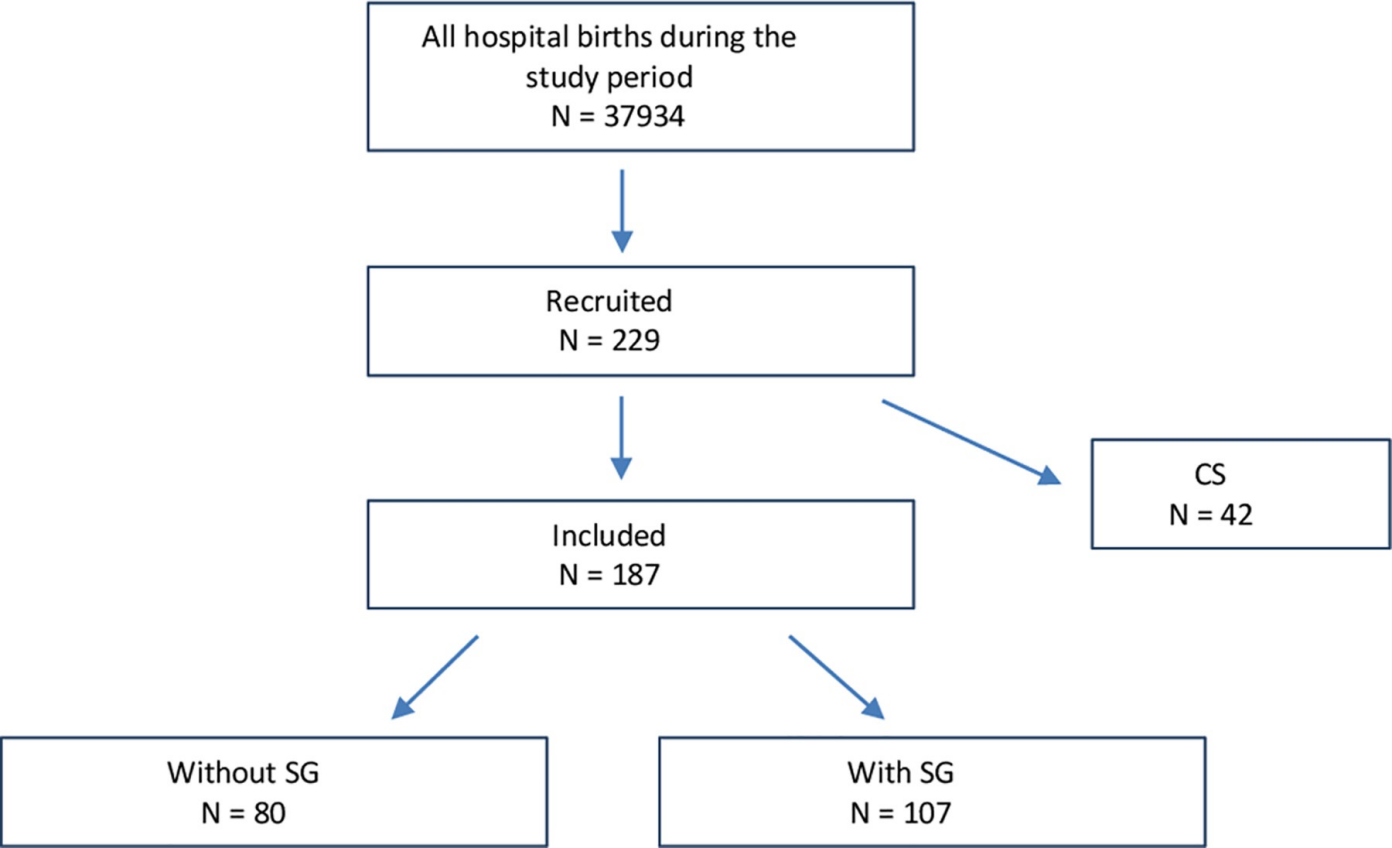
Study population.

Overall, 229 parturients were recruited during the study period, 42 (18%) were excluded because they underwent a cesarean section (Fig 1), and a total of 187 women were finally included. Of those, 107 (57%) women had SG and comprised the study group, while 80 (43%) did not have SG and comprised the control group. In addition, a subgroup analysis was carried comparing the demographical and clinical characteristics between the study group (those who had a vaginal birth) and those who were excluded (CS); no significant differences were noted between the groups.

The demographic and clinical characteristics of the study groups are presented in Table 1.

**Table 1:** Demographic and clinical maternal characteristics of the study groups stratified by the presence of SG.

		No SG = 80	SG = 107	
Maternal age (Mean±SD)		31.06±5.04	28.83±5.34	<0.01
Ethneicity n (%)	Jewish	63 (78.7%)	91 (85%)	0.34
	Bedouins	17 (21.3%)	16 (15%)	
BMI (Mean±SD)		27.50±4.20	30.98±6.05	<0.01
Chronic hypertension n (%)		2 (2.5%)	0 (0.0%)	0.10
Diabetes Mellitus n (%)		0 (0.0%)	1 (0.9%)	0.39
Preeclamsia n (%)		1 (1.3%)	0 (0.0%)	0.25
GDM n (%)		6 (7.5%)	8 (7.5%)	0.10
Gravidity (Median, IQR)		2 (1–3)	3 (2–4)	0.16
Parity (Median, IQR)		1 (0–2)	1 (0–2)	0.01
Previous Cesarean Section n (%)		5 (6.3%)	3 (2.8%)	0.25
Nulliparity n (%)		30 (37.5%)	28 (26.2%)	0.10
Grandmultiparity n (%)		3 (3.8%)	5 (4.7%)	0.53

SG—striae gravidarum; BMI—Body Mass Index; GDM—Gestational Diabetes Mellitus; IQR–interquartile range

Women with SG were significantly younger (28.83 vs. 31.06 years, p<0.01) and had a higher body mass index (30.98 vs. 27.5, p<0.01). Women with SG were less likely to conceive following artificial reproductive treatment. Delivery characteristics are presented in Table 2; mode of delivery and gestational age were comparable between the groups. Women with SG gave birth to larger neonates, which was statistically significant (3389 vs. 3155 grams, p<0.01). The differences in rates of perineal tears between women with and without SG are presented in Table 3. Women with SG were further sub-divided by severity, as displayed in Table 4. Eighty women (42.7%) did not have SG, 25 (13.3%), 43 (23%), and 39 (20.9%) women had mild, moderated, and severe SG. After delivery, 71 (38%) women had suffered from 1^st^ or 2^nd^-degree perineal tears, and none had 3^rd^ or 4^th^-degree perineal tears. Thirteen (7%) women underwent an episiotomy and therefore were not included in any of the two ’perineal tears’ groups. No significant differences were found in rates of perineal tears between women with and without SG (P = 0.91), regardless of SG severity (P = 0.38) (Tables 3 and 4).

**Table 2:** Delivery characteristics of the study groups stratified by the presence of SG.

		No SG = 80	SG = 107	
Gestational age at delivery (Mean±SD)		39.53±1.80	39.94±1.37	0.08
Birth weight (Mean±SD)		3155.76±511.40	3389.54±453.94	<0.01
Oligohydramnios n (%)		0 (0.0%)	4 (3.8%)	0.08
Mode of delivery n (%)	CS*	0 (0.0%)	2 (1.9%)	0.63
	Spontaneous VD	78 (97.5%)	101 (94.4%)	
	Operative VD	2 (2.5%)	4 (3.7%)	
Epidural analgesia n (%)		55 (69.6%)	70 (65.4%)	0.55

*2 cases of CS were included due to failed Vacuum extraction

SG—Striae gravidarum; CS–cesarean section; VD–vaginal delivery

**Table 3:** Differences in rates of perineal tears between women with and without SG.

	No SG = 80	SG = 107	
No perineal tear	43 (53.8%)	60 (56.1%)	0.72
Episiotomy n (%)	6 (7.5%)	7 (6.5%)	0.80
Grade 1/2 perineal tear n (%)	31 (38.8%)	40 (37.4%)	0.91
Grade 3/4 perineal tear n (%)	0 (0.0%)	0 (0.0%)	NA

**Table 4:** Differences in rates of perineal tears between women with and without SG, divided by severity.

	No striea (n = 80)	Mild (n = 25)	Moderate (n = 43)	Severe (n = 39)	P value
Grade 1/2 perineal tear n (%)	31 (38.7%)	6 (24.0%)	20 (46.5%)	14 (35.9%)	0.38
Grade 3/4 perineal tear n (%)	0 (0.0%)	0 (0.0%)	0 (0.0%)	0 (0.0%)	NA

We conducted a separate post-hoc analysis stratifying the patients according to the outcome of perineal tears. Demographical and clinical maternal characteristics of the study groups stratified by the presence of perineal tears are presented in S1 Table. Patients with no perineal tears were more likely to be of a higher gravidity and parity order and a higher rate of grandnultiparous women were in this group. In contrast women with perineal tear were more likely to be nulliparous. No other differences in baseline demographical and clinical characteristics were noted between the groups. Delivery characteristics of the study groups stratified by the presence of perineal tears are presented in S2 Table. Apart from a higher rate of epidural analgesia in the group of perineal tears no other differences were noted between the groups in delivery characteristics.

## Discussion

The primary purpose of this study was to investigate whether the prevalence of SG during late pregnancy is associated with perineal lacerations at the time of vaginal delivery. Our study’s main finding was that the presence of SG was not associated with perineal tears during labor. Halperin et al. [10]. conducted a similar study which showed an association between SG and perineal tears. However, in contrast to our study, their study evaluated SG after delivery.

Furthermore, their study used a different scoring method to assess SG severity. The technique used by Halperin et al. was developed by Atwal, Manku, Griths, and Polson [16] and provided a score based on observation of four areas (hips, buttocks, breasts, and abdomen) in primiparae [10]. We chose to use the Davey Scoring system, which assesses SG on the abdomen alone, thus decreasing confirmation bias. Furthermore, the Davey Scoring System is suited for primiparas and multiparas, thus avoiding selection bias and increasing sample size. Wahman et al. [17], conducted a similar study which found that abdominal stretch may serve as a predictor for vaginal lacerations when controlling for episiotomy. However, their study had a smaller sample size, and the number of patients who did not have an episiotomy was too small to show any statistically significant association. In contrast to our study, they did not use a well-developed scoring system to assess SG, and their method for evaluating stretch marks relied solely on the number of stretch marks. Like Halperin et al. [14], they too assessed SG after delivery.

Previous studies reported that maternal age, weight gain during pregnancy, and large fetal weight also serve as risk factors for the occurrence of SG [1, 7, 16, 18]. Those findings were in accordance with our results. The severity of SG is classified by number, color, and area of striae [3, 16]. However, the severity of SG, while very concerning for patients, is not considered a medical condition that requires routine follow-up [19]. Our study supports this practice.

The risk factors for the development of perineal trauma include maternal age, parity, gestational age, birth weight, fetal position, and maternal position [8, 16, 18, 20]. Literature assessing the role of SG on perineal trauma is scarce. Furthermore, to the best of our knowledge, the few previous studies in the field have not excluded women who underwent an episiotomy.

The molecular pathogenesis of SG remains unclear, with various components of the dermal extracellular matrix implicated. The extracellular matrix provides the skin with elasticity and strength [12]. The skin elasticity is provided mainly by elastin, and strength is primarily provided by type I collagen fibers. When comparing SG to normal skin histologically, there is decreased extracellular matrix and collagen, as well as atrophy and loss of rete ridges [2]. That being said, not all SG are equal, and the development or severity of SG depends mainly on the affected individual rather than the circumstances involved. Our study holds several advantages. First and foremost, to the best of our knowledge, this is the first study to assess the association between SG and lacerations without considering patients who underwent an episiotomy. In addition, this study is the only study to evaluate SG severity before delivery.

The study took place at the Soroka University Medical Center, which is the sole tertiary center for the population of southern Israel. This fact allowed us to avoid selection bias, recruit a heterogeneous population and increase the generalizability of our findings.

Although our study population included Jewish and Bedouin women but not other ethnicities, it could be argued that our conclusion is too generalized. We are well aware of racial and ethnic disparities in adverse perinatal outcomeswe believe that in Israel the situation is different allowing us to make assumptions regarding the generalizability of our findings [21]. Israel contains a variety of immigrants at various stages of cultural assimilation. Marked differences have been observed between Jewish immigrants from Europe, the US, North Africa, and the Middle East who, although are all Jewish, differ in cultural and genetic backgrounds and therefore represent a variable and diverse population [22]. Unfortunately we do not have information regarding the ethnic background of our population.

In addition, all data was directly recorded from each patient’s file and updated online, double-checked by multiple staff members, allowing very little room for mistakes and missing data. Finally, we used the Davey Scoring System to assess SG, a simple and easy method to use, leaving little room for error.

Nonetheless, the study had some limitations. The Davey Scoring system was the only tool used to measure SG severity. In this study, we did not conduct biopsies to characterize SG severity histologically. However, conducting an invasive approach in this study would likely impede consent. The Davey Scoring system is a validated, commonly used study tool for both pregnant and non-pregnant patients [3, 6].

## Conclusion

In conclusion, we have demonstrated that women with SG were significantly younger and had a higher body mass index. We also found that women with abdominal SG gave birth to bigger neonates and this difference was statistically significant. Abdominal SG does not seem to increase the risk for spontaneous perineal tears. Future prospective studies should evaluate the pathogenesis of SG. Women with abdominal SG, regardless of severity, can be reassured that they are not at an increased risk for perineal lacerations.

## Supporting information

S1 Table Demographic and clinical maternal characteristics of the study groups stratified by the presence of perineal tears. BMI—Body Mass Index; GDM—Gestational Diabetes Mellitus; IQR–interquartile range.
(DOCX)

S2 Table Delivery characteristics of the study groups stratified by the presence of perineal tears. *2 cases of CS were included due to failed Vacuum extraction. CS–cesarean section; VD–vaginal delivery.
(DOCX)
