# Supplementary material for: The association between striae gravidarum and perineal lacerations during labor
Source: PLoS One. 2022 Mar 15;17(3):e0265149. doi: 10.1371/journal.pone.0265149 (PMC8923500; doi:10.1371/journal.pone.0265149)
Supplement: S1 Table — BMI—Body Mass Index; GDM—Gestational Diabetes Mellitus; IQR–interquartile range. (DOCX) [file pone.0265149.s001.docx]

|  |  | Perineal tear=71 | No perineal tear/Isolated episiotomy=116 | P value |
| --- | --- | --- | --- | --- |
| Maternal age (Mean±SD) |  | 29.09±5.27 | 30.20±5.34 | 0.16 |
| Ethneicity n (%) | Jewish | 62 (87.3%) | 92 (80.0%) | 0.20 |
|  | Bedouins | 9 (12.7%) | 23 (20.0%) |  |
| BMI (Mean±SD) |  | 29.29±6.16 | 29.60±6.16 | 0.31 |
| Chronic hypertension n (%) |  | 1 (1.4%) | 1 (0.9%) | 0.72 |
| Diabetes Mellitus n (%) |  | 0 (0.0%) | 1 (0.9%) | 0.43 |
| Preeclamsia n (%) |  | 0 (0.0%) | 1 (0.9%) | 0.43 |
| GDM n (%) |  | 2 (2.8%) | 12 (10.3%) | 0.06 |
| Gravidity (Median, IQR) |  | 2 (1-3) | 3 (2-4) | <0.01 |
| Parity (Median, IQR) |  | 1 (1-2) | 3 (2-4) | <0.01 |
| Previous Cesarean Section n (%) |  | 4 (5.6%) | 4 (3.4%) | 0.47 |
| Nulliparity n (%) |  | 33 (46.5%) | 25 (21.6%) | <0.01 |
| Grandmultiparity n (%) |  | 0 (0.0%) | 8 (6.9%) | 0.02 |
