# Supplementary material for: The association between striae gravidarum and perineal lacerations during labor
Source: PLoS One. 2022 Mar 15;17(3):e0265149. doi: 10.1371/journal.pone.0265149 (PMC8923500; doi:10.1371/journal.pone.0265149)
Supplement: S2 Table — *2 cases of CS were included due to failed Vacuum extraction. CS–cesarean section; VD–vaginal delivery. (DOCX) [file pone.0265149.s002.docx]

|  |  | Perineal tear=71 | No perineal tear/Isolated episiotomy=116 | P value |
| --- | --- | --- | --- | --- |
| Gestational age at delivery (Mean±SD) |  | 39.84±1.31 | 39.72±1.72 | 0.60 |
| Birth weight (Mean±SD) |  | 3275.75±460.88 | 3298.88±511.68 | 0.74 |
| Oligohydramnions n (%) |  | 2 (2.8%) | 2 (1.7%) | 0.63 |
| Mode of delivery n (%) | CS* | 0 (0.0) | 2 (1.7%) | 0.72 |
|  | Spontenous VD | 70 (98.3%) | 109 (94.0%) |  |
|  | Operative VD | 1 (1.7%) | 5 (4.3%) |  |
| Epidural analgesia n (%) |  | 58 (83.1%) | 68 (58.6%) | <0.01 |
